# Capability Sensitive Design for Health and Wellbeing Technologies

**DOI:** 10.1007/s11948-020-00275-5

**Published:** 2020-11-18

**Authors:** Naomi Jacobs

**Affiliations:** Department of Philosophy and Ethics, and Human-Technology Interaction, Eindhoven University of Technology, Eindhoven, The Netherlands

**Keywords:** Value sensitive design, Capability approach, Capability sensitive design, Design framework, Ethics by design, Ethics

## Abstract

This article presents the framework Capability Sensitive Design (CSD), which consists of merging the design methodology Value Sensitive Design (VSD) with Martha Nussbaum's capability theory. CSD aims to normatively assess technology design in general, and technology design for health and wellbeing in particular. Unique to CSD is its ability to account for human diversity and to counter (structural) injustices that manifest in technology design. The basic framework of CSD is demonstrated by applying it to the hypothetical design case of a therapy chatbot for mental health. By applying CSD to a design case, the merits of this new framework over the standard VSD approach become apparent. Also, the application demonstrates what a technology design would look like when attention is paid to capabilities right from the start of the design process.

## Introduction

In recent years, there has been an increasing awareness of the impact that technology design can have on supporting or undermining values.^1^ This awareness that technology design is not value-neutral, but instead embodies moral choices, has led to the development of various design methods that explicitly pay attention to values and ethical considerations. One of the most prominent and influential methods is Value Sensitive Design (VSD): a design methodology that aims to address and account for values in a "principled and systematic manner throughout the technical design process" (Friedman and Hendry 2019, p. 4). What is unique about VSD is that it proactively integrates ethics into technology design (Van den Hoven 2008).

Despite being a highly promising approach to ethics of technology design, VSD faces various challenges (for a detailed discussion of these challenges see:Borning and Muller 2012; Davis and Nathan 2015; Jacobs and Huldtgren 2018; Manders-Huits 2010;). The three most prominent challenges that VSD faces include: (1) obscuring the voice of its practitioners and thereby claiming unfounded moral authority; (2) taking stakeholder values as leading values in the design process without questioning whether what *is* valued by stakeholders also *ought* to be valued; and (3) not being able to provide normative justification for making value trade-offs in the design process (Jacobs and Huldtgren 2018; Manders-Huits 2010). To overcome these challenges, Noemi Manders-Huits (2010) and myself and Alina Huldtgren (Jacobs and Huldtgren 2018) have argued that VSD practitioners need to complement VSD with an ethical theory.^2^ The claim made by Manders-Huits (2010) and Jacobs and Huldtgren (2018) that VSD needs to be complemented by an ethical theory is accepted at face value in this paper. However, it is important to note that there may be other solutions to the challenges of VSD not requiring complementation of an ethical theory, like e.g. the ‘Sen-procedural-VSD-approach’ that Alessandra Cenci and Dylan Cawthorne (2020) argue for. However, this article focuses on providing the best possible case for and elaboration of complementing VSD with Martha Nussbaum’s substantial capability theory^3^ (2000, 2006, 2011), leaving the comparison with the Sen-procedural-VSD-approach (Cenci and Cawthorne 2020) to be dealt with in later work. By complementing VSD with Nussbaum’s capability theory the article contributes to the further development of VSD in particular, and the domain of ethics of technology design in general.

Various scholars have explored the role of the capability approach (CA) for ethics of technology and for designing for values (e.g. Cawthorne and Cenci 2019; Cenci and Cawthorne 2020; Coeckelbergh 2009, 2010; Frediani and Boano 2012; Haenssgen and Ariana 2017; Hancke 2016; Johnstone 2007; Mink et al. 2014; Murphy and Gardoni 2012a, 2012b; Nichols and Dong 2012; Oosterlaken 2013, 2015a; 2015b; Steen 2016; Zheng 2007, 2009). This article contributes to those efforts by providing a systematic investigation of how VSD’s tripartite methodology can be combined with the normative foundation of Nussbaum's capability theory -resulting in ‘Capability Sensitive Design’ (CSD)—and providing insight and tools for designers and engineers to use to operationalize CSD.

Ilse Oosterlaken (2012; 2013; 2015a; 2015b) has taken on a similar endeavor, taking a capability approach to technology design and indicating this as ‘Capability Sensitive Design’. However, Oosterlaken’s endeavor differs from this present endeavor because Oosterlaken examines various approaches in the domain of ‘designing for values’ broadly conceived, while this present endeavor focuses on ‘value sensitive design’ (VSD) (Friedman and Hendry 2019) explicitly.

CSD is intended to be useful for ethicists of technology in general, and for designers and engineers working on health & wellbeing technology in particular. CSD is particularly well suited to ethically evaluate technology design for health and wellbeing for three reasons. Firstly, the primary focus of CSD on people's capabilities fits very well with the common aim of technology design to enhance and expand what people are able to be and do (Oosterlaken 2012; Van den Hoven 2012). Secondly, by putting focus on conversion factors, i.e. people's abilities to *converse* resources into capabilities, CSD is able to account for human diversity (Oosterlaken 2012; 2015a; Toboso 2010; 2011). Why this is important is elaborated on in more detail in "Capability Approach and Capability Theory" section. Thirdly, CSD is particularly well suited to the domain of technology design for *health and wellbeing* because CSD aims to normatively assess technology design based on whether the design expands human capabilities that are identified as valuable. Lennart Nordenfelt (1986) and Sridhar Venkatapuram (2013) have proposed to conceptualize health as a person's ability to realize one's vital goals and to achieve or exercise a cluster of basic human activities. Given that we adhere to this conception of health, then CSD seems to be particularly suited to normatively assess technology designs for health and wellbeing.

The article proceeds as follows: first, the distinction between the capability *approach* and capability *theories* is elaborated on together with the core elements that all capability theories share. Secondly, the specifics of CSD are discussed. Thirdly, CSD is applied to a hypothetical design case of technology for health and wellbeing: the design of a therapy chatbot to help improve people's mental health. This basic outline of CSD illustrates (1) the general CSD framework (2) how CSD can be applied to a (hypothetical) health and wellbeing technology design such as a therapy chatbot, and (3) what the merits are of a CSD analysis over the standard VSD approach for health & wellbeing technology design. To conclude, the main challenges that CSD faces are discussed, followed by how CSD could address these.

## Capability Approach and Capability Theory

Since the mid-1940s, the dominant way to measure the overall welfare of a country has been to look at Gross Domestic Product (GDP) or Gross National Product (GNP) per capita (Dickinson 2011). Sen (1985), however, famously challenged this dominant focus by illustrating empirically how deceiving GDP or GNP per capita can be as measures of wellbeing. Instead of looking at GDP or GNP to measure wellbeing, the economist and pioneer of the capability approach, argued that one should look at what people are able to do and be, and thus the kind of life that they are effectively able to lead, when assessing people's well-being. That is because quantities of or access to resources or goods don't tell us much about what a person is actually able to be and do in one’s life and the real opportunities available to a person (Sen 2009). A visually impaired and a visually non-impaired person, for example, would, in a given context need different amounts and types of resources to enable them to have the same opportunities in life. Thus, although resources are necessary *means* to wellbeing and freedom, we should look at the freedoms and opportunities people have available to them when assessing human wellbeing.

Before elaborating on some of the key characteristics of the capability approach, it is important to briefly clarify the distinction between the capability *approach* and capability *theories* as put forward by capability scholar Ingrid Robeyns (2017). Robeyns explains that the capability approach is an "open-ended and underspecified framework, which can be used for multiple purposes" (2017, p. 29). As an open and general idea, the capability approach can be specified or 'closed' in many ways. When one specifies the approach and puts it to a specific use for a particular purpose, Robeyns proposes to speak of a capability *theory*. In short: there is one capability approach and there are many capability theories (Robeyns 2017).

My aim in this article is to specify the open-ended and underspecified framework and put it to use for a specific purpose, namely to ethically evaluate the design of health and wellbeing technologies. The capability *theory* that follows is called 'Capability Sensitive Design' (CSD) and consists of a combination of the method of VSD, combined with the core elements from the general capability approach and the normative foundation from the specific capability theory developed by Nussbaum (2000; 2006; 2011).

The purpose of CSD is to normatively evaluate the design of health and wellbeing technology, accounting specifically for human diversity and diminishing (structural) injustices in technology design. CSD is intended to be useful for ethicists of technology in general, and in particular for designers and engineers working on health & wellbeing technology who aim to design with moral sensitivity. Before elaborating further on the specific capability theory of CSD, let me briefly discuss the core elements that *all* capability theories share as put forward by Robeyns (2017).

*Functionings* and *capabilities* are the core concepts of every capability theory. Capabilities refer to people's freedoms and the valuable opportunities that one can choose from. Functionings refer to what people are *actually* achieving in terms of beings and doings. For example, the opportunity to travel is a capability, while actually travelling is a functioning. Thus, functionings are about the *actually realized*, while capabilities are about the *effectively possible* (Robeyns 2017). There are functionings and capabilities with positive value, as well as negative value (e.g. committing murder). In themselves, functionings and capabilities are neutral concepts. It is subsequently a normative decision of the *specific* capability theory to decide *which* capabilities are defined as having positive value.

Another core element is the idea that it should not be taken for granted that resource provision leads to increased capabilities or functionings. Instead, the *conversion* of goods and services into what people are actually able to be and do is influenced by personal, social, and environmental *conversion factors*. Conversion factors "determine the degree to which a person can transform a resource into a functioning" (Robeyns 2017, p. 45).

There are three different types of conversion factors: (1) *personal conversion factors* are internal to a person, such as metabolism, physical condition, sex, or intelligence. (2) *Social conversion factors* stem from the society in which one lives, such as public policies, social norms, societal hierarchies or power relations related to class, gender, race or caste. (3) *Environmental conversion factors* emerge from the physical or built environment that a person lives in (Robeyns 2017, p. 46).

Think for example of a woman who buys a wearable fitnesstracker to help increase her capability of bodily health. This woman could have the personal conversion factor of having a sufficient physical condition to be able to walk and run and in that way use the fitnesstracker. She might also have the right environmental factors needed, such as having broad sidewalks and a park nearby to exercise in. But she might lack the social conversion factor needed if she lives in a neighborhood where it is unsafe for women to go out on their own. Differences in conversion factors form an important source of human diversity, which is a notion of great importance within the capability approach and CSD. The notion of human diversity is discussed in more detail at the end of this section.

Furthermore, it is important to always be clear whether one values something as an end in itself, or as *a means to* a valuable end. Within each capability theory, the capabilities that are defined as valuable are the ultimate ends. Subsequently, normative decisions are made based on the extent to which something -e.g. policies or design choices- promotes people's capability to achieve the functionings they value. However, functionings and capabilities are not necessarily the only dimensions of value, many capability theories may add additional dimensions of value, such as fairness. The fact that there might be other dimensions of value besides capabilities and functionings, indicates *value pluralism*. Another dimension of value pluralism is the fact that there are multiple capabilities and functionings to be valued, rather than just one. A final core element of every capability theory is that every human being has equal moral worth (Robeyns 2017).

Now that the core elements of all capability theories as put forward by Robeyns (2017) are mentioned, it is important to note that these key insights have often been compared to (rival) ethical theories such as e.g. utilitarian views, the manifold ethical–political theories that pervade contemporary liberal-egalitarian thinking such as John Rawls’ (1971) theory of justice, or other multidimensional accounts of wellbeing-related needs such as the account of Doyal and Gough (1984). To discuss these comparisons in depth here would exceed the limits of this paper but I would like to refer to the works of e.g. Gough (2014), Robeyns (2009), Brighouse and Robeyns (eds.) (2010) in which in depth comparisons are made.

## Capability Sensitive Design

The abovementioned core elements are shared by all capability theories. It is now time to clarify the specifics of the capability theory of CSD. The specific purpose of CSD is to ethically evaluate the design of health and wellbeing technologies. CSD consists of a combination of the method of VSD, combined with the normative foundation from the specific capability theory developed by Nussbaum (2000; 2006; 2011).

The reason why Nussbaum’s capability theory is chosen to complement VSD (and not for example Sen’s procedural capability account (Sen 1992; 1999; 2009)) is because VSD faces various challenges that have to do with the fact that VSD is a procedural approach that does not make any substantive ethical commitments (Manders-Huits 2010; Jacobs and Huldtgren 2018). VSD needs complementation of a substantive ethical theory that provides grounds of justification and argumentation for moral claims and considerations (Jacobs and Huldtgren 2018) because without such substantive ethical commitments, VSD faces the challenges of (1) obscuring the voice of its practitioners and thereby claiming unfounded moral authority; (2) taking stakeholder values as leading values in the design process without questioning whether what *is* valued by stakeholders also *ought* to be valued; and (3) not being able to provide normative justification for making value trade-offs in the design process (Jacobs and Huldtgren 2018; Manders-Huits 2010). Nussbaum’s capability theory provides such needed substantive normative foundation by defending that all people are morally equal and deserve a life worth living, which entails that every human being should have access to ten central capabilities. By explicitly complementing VSD with this substantive normative foundation of Nussbaum’s capability theory, the CSD framework is able to provide sources of justification and argumentation for moral claims and considerations, which are needed to make principled judgments, to attend to a set of bounded and principled values, and to avoid conflating facts with values (Jacobs and Huldtgren 2018). Nussbaum’s capability theory helps VSD to overcome the challenge of obscuring the voice of its practitioners as well as the naturalistic fallacy, this will be elaborated on in further detail in the section ‘Conceptual Investigation’ of this article. Important to note, as already indicated in the introduction, is that e.g. Cenci and Cawthorne (2020) have presented a solution to the challenges of VSD not requiring complementation of a substantial ethical theory, arguing instead for a ‘Sen-procedural-VSD-approach’. This article, however, leaves the comparison with the Sen-procedural-VSD-approach (Cenci and Cawthorne 2020) to be dealt with in later work and instead focuses on providing the best possible case for and elaboration of complementing VSD with Nussbaum’s substantial capability theory. Let us now look further into the normative foundation of Nussbaum’s capability theory.

Nussbaum has developed a partial theory of justice based on the moral assumption that all people are morally equal and deserve a life worth living. Nussbaum identifies ten capabilities that should be available to all human beings. Nussbaum defends these ten capabilities as being the moral entitlements of every human being. The ten capabilities are:

1. being able to live a normal length of lifespan;
2. having good health;
3. maintain bodily integrity;
4. being able to use the senses, imagination, and think;
5. having emotions and emotional attachments;
6. possess practical reason to form a conception of the good;
7. have social affiliations that are meaningful and respectful;
8. express concern for other species;
9. able to play;
10. have control over one’s material and political environment (2000, p. 33).

Important to note here is that Nussbaum’s list of capabilities is often criticized as paternalistic or illegitimate with the reason that such a list of capabilities should be the outcome of a democratic process or a process of public reasoning (Cenci and Cawthorne 2020; Claassen 2011). Making a capability list as Nussbaum does -and upon which CSD relies- would bypass those people that its theory is to be applied to in practice, one could argue (Claassen 2011). A related critique is that a philosopher such as Nussbaum cannot possibly know which capabilities are important to people because there are epistemological limits to the philosopher's insights, and thus claiming false authority with a list (Claassen 2011). In response to these points of criticism, Nussbaum deliberately formulated the ten capabilities on her list at an abstract level, ‘precisely in order to make room for the activities of specifying and deliberating by citizens’ (Nussbaum 2000, p. 79). She emphasizes that the translation of the capabilities into implementations, policies -or in this context: design requirements- should take into account differences on the specific, local level.^4^ The capabilities from Nussbaum's list thus need to be specified in a particular context. Thus, the CSD practitioners together with the stakeholders involved provide context-specific specification of the capabilities, adding content to the abstract capabilities on the open-ended list. Furthermore, Nussbaum emphasizes that her list of ten capabilities is not definite; instead, the list is open for discussion and revision (Nussbaum 2000, p. 77). Thus, Nussbaum’s list offers a piece of input into the deliberation process, providing us with a stimulus for public debate, capability-scholar Claassen (2011, p. 501) points out. Now, a preferred strategy for CSD practitioners to make use of Nussbaum’s capability list is put forward by Claassen (2011) as the “philosopher-investigator” approach:

philosopher-investigators regularly cross the boundaries to gather data in the ‘real world’ and they may learn from public debates about capabilities, or they may even conduct social scientific research to find out which capabilities people value most. They will retain their right to construct their theories as they deem best; for after all, the fact that a group of people empirically holds the belief that realizing capability x is a moral demand does not mean that these beliefs are morally correct. However, with suitable modification, they will allow themselves to let the results of their practical investigations influence their theories and think the latter enriched by these efforts (Claassen 2011, pp. 504–505).

A good example of this strategy is the research done by Jonathan Wolff and Avner de-Shalit, who used Nussbaum’s capability list as the basis for in-depth interviews and then modified the list to account for the results of these interviews (Wolff and de-Shalit 2007). Thus, the philosopher-investigator’s strategy provides an answer to the objection that Nussbaum’s list of ten capabilities might be illegitimate, paternalistic or claiming false moral authority because the list is not the mere result of isolated reflection but is also informed by empirical study and stakeholder analysis, thus drawing upon the knowledge of (many) others. A practical tool to assist empirical stakeholder analysis could be the use of a set of “capability cards” as developed by Marc Steen (2016) which provides input to support CSD practitioners and stakeholders to discuss, select and specify the capabilities (from Nussbaum’s list) on which to focus specifically in the design context at hand.

Nussbaum’s list of ten capabilities forms the normative foundation of CSD together with two moral principles: respecting human diversity and diminishing (structural) injustices in technology design. Let me now briefly elaborate on these principles.

### Human diversity and (structural) injustices in technology design

Each individual has a unique profile of conversion factors, some of which are body-related, while others are shared with all people from one’s community, and still others are shared with people with the same social characteristics, e.g. same gender, class, caste, age, or race characteristics (Robeyns 2017, p. 113). Taking into account conversion factors -i.e. human diversity- in technology design is important in order to avoid potential unjust risks of harm for some user groups (Oosterlaken 2012).

Some technology is deliberately designed for a specific user group whose members share the same conversion factors, purposefully excluding users that do not share those conversion factors. This does not have to be problematic, think e.g. of the design of a bicycle for children that is designed to fit the size of boys and girls around the age of 4 to 6 years old. Most people younger than 4 years-old or older than 6 years will have body-related conversion factors that will not enable them to make use of the particular bicycle design. This is fairly unproblematic since the design is particularly intended to serve a specific user group (children between 4 and 6-years old), taking into account their specific conversion factors.

However, what I want to focus on here is a much more subtle difficulty concerning human diversity in technology design. That is the difficulty that, even though designers might not purposefully exclude users from their design, by designing technology that is in principle intended for a (large) user group but that not actually serves all members of that user group equally because certain conversion factors of people are not sufficiently taken into account, designers may increase a risk of harm for some users to which designers are (partially) responsible (Oosterlaken 2012). Let me discuss two examples.

Recently, the design of a soap dispenser in a public restroom caused astonishment when it turned out that the machine only worked on white skin (Lazzaro 2017). The designers of the soap dispenser failed to take into account that the infrared sensor of the dispenser could not detect darker skin tones. Although the designers did not purposefully exclude people with darker skin tones, the designers did not sufficiently consider the bodily conversion factor of people with darker skin tones, even though the soap dispenser in principle was designed for people of *all* skin tones. Failing to account for the bodily conversion factor of people with darker skin tones, the design has (unintendedly) put these users at an increased risk of harm.^5^

Another example includes the design of cars; in comparison to men, women tend to sit further forward when driving because women are on average shorter than men. Women’s legs -on average- needs to be closer to reach the pedals and they need to sit more upright to see clearly over the dashboard. However, this is not the ‘standard seating position’ for which cars are designed. Research by Caroline Criado-Perez (2019) has shown that as a result, women are at greater risk of injury when involved in a car crash than men. Both of these examples show that although a technology is designed for a large user group (respectively including people of *all* skin tones and men *as well as* women) the technology design fails to sufficiently take into account the relevant conversion factors of all the people in that user group. With the result that these people (i.e. respectively people with darker skin tones and women) are not able to convert the technology into a mean (as is the case with the soap dispenser for people with darker skin tones) or are at an increased risk of injury when making use of the technology (women drivers). The failure to sufficiently account for the conversion factors of all the users in your intended user group is to a large extend a blame on part of designers, and it is their responsibility to avoid such injustices occurring in technology design by paying more attention to human diversity, i.e. the relevant conversion factors of all intended users.

Mackenzie (2013) has drawn a strong connection between human diversity, capability deficits, and vulnerability. Mackenzie argues that specific capability deficits can signal sources of vulnerability, and vice versa, since "people's ability to convert the resources available to them into achieved functionings will vary according to […] individual differences and external circumstances" (2013, p. 50). For example, a woman who is a lesbian, may have the capacity for sexual expression but will not develop the capability to do so if she is a member of a community that strongly condemns same-sex relationships. Furthermore, Mackenzie argues that the notion of vulnerability "signals the actual or potential harm that may result from particular capability deficits and highlights the obligation to address these deficits in order to remediate vulnerability" (2013, p. 50). The lesbian woman who does not have the capability to express her sexuality is vulnerable to a range of possible harms, such as social ostracism, a decrease in employment opportunities, or being a victim of homophobic violence. If we think that the capability of sexual expression is important,^6^ then this capability deficit and the woman's vulnerability to aforementioned harms are unjust and call for action to remediate her vulnerability, Mackenzie rightfully argues (2013, p. 51).

Elsewhere I have argued that technology design should explicitly consider the needs and interests of vulnerable people (Jacobs 2020). That is because vulnerability is to be understood as a diminished capacity to meet or protect ones needs or safeguard one’s interests, which could lead to an increased likelihood of suffering harm or wrong (Jacobs 2020).

Oosterlaken, who has worked extensively on the relation between technology and capabilities (2013), has pointed out that the key is to realize that if a technology is designed in such a way that for some (vulnerable) user groups the technology will not expand their capabilities, the technology design might be morally unjust (Oosterlaken 2013). CSD endorses the ten capabilities identified by Nussbaum (2000) as to have moral value and people should be brought to at least a threshold level of these capabilities to lead a dignified life. Now, if a technology design fails to bring a particular stakeholder group to the threshold level of one or more of these capabilities, then the technology design is not only inadequate but could also be morally unjust. In other words: CSD is able to signal whether there is a (structural) injustice at play in a technology design when a particular stakeholder group for whom the technology is (partly) intended is not being brought up to the threshold level of one or more capabilities that have been identified to have moral value in the particular design context.

Think for example of an architectural design of a town hall, which is a public space that is intended to be public for all, that is designed with a two-step stairway in front of the entry and a 3-inch-high doorsill at the entry door. For people who are in a wheelchair, the stairway and doorsill make the town hall inaccessible to them. For people who have difficulty walking, such as people who walk with crutches or elderly who walk with a walking stick, the town hall is difficult to access. For people who have no difficulty walking, the town hall is easily accessible. CSD endorses the ten capabilities from Nussbaum's list to have moral value to all people in order for them to live a dignified and worthy life. Now, one of the capabilities from Nussbaum's list that is relevant in this context is the capability of having control over one's environment and being able to effectively participate in political and social choices that govern one's life. We now see that people who have no difficulty walking can easily access the town hall, a place where political and social choices are made and where one can effectively enact on controlling one's environment. The design of the town hall thus enhances the capability of having control over one's environment for people who have no difficulty walking. For the people who are in need of walking assistance such as crutches or walking sticks, the town hall is more difficult to access and we see that for these people it is harder to reach the threshold level of being able to have control over one's environment for people. And for the people who are in a wheelchair, the town hall is entirely inaccessible which makes it impossible for these people to effectively govern their environment by ways of political or social choices that are made in the town hall. The design of the town hall thus fails to bring these people to the threshold level of the capability to control one's environment. This makes the design of the town hall not only inadequate, but also indicates there is an injustice at play in the design. That is because the design of the town hall fails to bring a particular vulnerable stakeholder group (i.e. people who experience difficulty with walking), for whom the town hall is also intended, to the threshold level of the capability of having control over one's environment and being able to effectively participate in political and social choices that govern one's life, which is a capability that is identified to have moral value.

## CSD for Health & Wellbeing Technology

Up to this point, the article has reflected on why CSD could be well suited to ethically evaluate technology design in general. In this section, I briefly elaborate on the reason why CSD is well suited to ethically assess technology design for *health and wellbeing*.

Health has been conceptualized in various ways, most prominently as: (1) absence of disease (Boorse 1975); as (2) a state of complete physical, mental, and social well-being (WHO 2006); as (3) the ability to adapt and self-manage in the face of social, physical, and emotional challenges (Huber and colleagues 2011); as (4) the ability to realize one's vital goals (Nordenfelt 1986); and as (5) a person's ability to achieve or exercise a cluster of basic human activities or capabilities (Venkatapuram 2013). Not only do we have various conceptions of health, we also have a variety of health practices. Such as the health practices of biomedical research, care for chronically ill patients, palliative care, reproductive medicine, public policies concerned with health inequalities, or the practice of designing technology to enhance health and wellbeing. Given the variety of the purposes of different health practices, these different practices may need different health concepts. Haverkamp et al. (2018) have argued that "it makes sense to take different health concepts as appropriate for guiding different practices" (p. 382), instead of seeking one 'overall' conceptual theory of health that applies to *all* contexts and practices. Haverkamp et al. (2018) argue that if we want a health concept to be relevant for a certain health practice, such a health concept should guide the practice in "their formulation of and reflection on goals and priorities" (p. 382).

The practice of technology design to enhance health and wellbeing can be appropriately guided by the conceptions of health as formulated by Nordenfelt (1986) and Venkatapuram (2013), since these conceptions focus respectively on a person's ability to realize one's vital goals and a person's ability to achieve or exercise a cluster of basic human activities or capabilities, which corresponds well with technology design that aims to enable people's abilities. Consider technology designs ranging from glasses, hearing aids and prostheses that enable and enhance people's abilities, to technology designs such as online applications that help people achieve or maintain a healthy lifestyle (e.g. MyFitnessPal or Fitbit), or manage their diabetes (MySugr) or their alcohol consumption (Sobriety Counter) and thereby enable them to achieve their goals and abilities.

Now, given that the conceptions of health by Nordenfelt (1986) and Venkatapuram (2013) are most suited to guide the practice of technology design for health and wellbeing, then CSD -based on the core elements of the capability approach and the normative foundation of Nussbaum's capability theory in particular- seems to be particularly well suited to normatively assess such technology designs for health and wellbeing.

## Applying Capability Sensitive Design

In what follows the workings of CSD are demonstrated. I propose that CSD follows the tripartite and iterative methodology of VSD, which entails a conceptual, empirical and technical investigation (Friedman and Hendry 2019). Each of these investigations is carried out iteratively, mutually informing and being informed by the other investigation. I will explicate what each of these investigations entails in CSD by discussing the example of a hypothetical design case of an AI-based therapy chatbot that aims to help people deal with mental health issues such as depression, dysfunctional thinking, or anxiety disorders. The following CSD outline illustrates: (1) the general workings of CSD, (2) what a new technology design for health and wellbeing such as an AI-based therapy chatbot would look like when attention is paid to capabilities right from the start of the design process, and (3) what the advantages are of a CSD analysis compared to the standard VSD approach.^7^

### Designing a therapy chatbot for mental health

Data from the World Health Organization shows that "globally, more than 300 million people of all ages suffer from depression" (WHO 2019). While at the same time 33% of countries worldwide "allocate less than 1% of their total health budgets to mental health, with another 33% spending just 1% of their budgets on mental health", furthermore "there is only one psychiatrist per 100 000 people in over half the countries in the world" (WHO 2001). Partly in response to these alarming data, conversational 'therapy chatbots' are being introduced into the domain of clinical psychology (Hoermann et al. 2017; Kretzschmar et al 2019).^8^ The aim of these therapy chatbots is to assist people in dealing with mental health issues such as depression, dysfunctional thinking, or anxiety disorders.

One of the first conversational chatbots introduced for mental health was the system called ELIZA developed by Joseph Weizenbaum (1966) that "emulated a Rogerian psychological perspective and used an early form of AI to engage the user in a dialogue of question and answer and asked empathic questions, based on the previous dialogue move" (Calvo et al. 2017). Present-day therapy chatbots, such as Woebot, Wysa and Youper, are now making use of natural language processing systems (NLPS). With machine learning algorithms for language processing, these therapy chatbots analyze and process large amounts of natural human language data, replicate patterns of human interactions and respond based on the knowledge database that is available to them at that point in time. Utilizing NLP techniques, these therapy chatbots deliver cognitive behavioral therapy (CBT) interventions to users. CBT is a therapy that modifies dysfunctional emotions, behaviors and thoughts. Online CBT-based interventions have been widely developed and evaluated effectively for the treatment of various mental health issues, such as depression, dysfunctional thinking, and eating or anxiety disorders (Barak and Grohol 2011; Calvo et al. 2017; Kaltenthaler et al. 2006). Online CBT interventions include for example daily 'check-ins' wherein the chatbot engages users in brief conversations asking how the user is feeling at that moment, or 'mood tracking' that enables users to track their moods with the help of emoticons or keywords over a period.

The aim of therapy chatbots that are making use of CBT interventions is to change the attitude and behavior of users for them to better cope with their mental problems such as dysfunctional thinking, depression or anxiety disorders. These therapy chatbots can therefore be understood to fall in the category of behavior change technologies (BCTs). BCTs are technologies intentionally designed to induce behavior change in users, making use of various behavior change techniques. Such behavior change techniques include e.g. suggestion; intervening with a signal or message at the right time (Fogg 2003, p. 41). For instance, if it becomes apparent trough the CBT intervention of mood tracking that a user is regularly feeling sad in the afternoons, then by intervening with the CBT intervention of a 'check-in' in the afternoon is the 'right time' to stir a change in behavior of the user. Another behavior change technique is self-monitoring: through self-tracking their behaviors and moods, users learn how well they are performing the target behavior, "increasing the likelihood that they will continue to produce the behavior" (Fogg 2003, p. 44). A third behavior change technique that is often used is that of conditioning: by rewarding target behavior the user is enforced "to increase the instances of a behavior or to shape complex behaviors" (Fogg 2003, p. 49). Woebot, for instance, sends users funny GIFs when they answer a question correctly.

### Conceptual Investigation

The aim of the conceptual analysis is to: (1) select the capabilities and corresponding functionings that are relevant in the particular design context, (2) get clear who the stakeholders are that are affected by the technology design, and subsequently (3) identify what the relevant conversion factors at play are for these stakeholders.

Nussbaum’s list of ten capabilities forms the starting point for the selection of relevant capabilities in the particular design context at hand. A practical tool to assist CSD practitioners in selecting relevant capabilities from the list could be a set of “capability cards” as developed by Steen (2016) which provides input to support CSD practitioners and stakeholders to discuss, select and specify the capabilities from Nussbaum’s list and help them decide upon which to focus specifically in the design context at hand.^9^ The precise specification of the selected capabilities in the technology design will continue to evolve in the empirical and technical investigations, since CSD follows the integrative and iterative methodology of VSD wherein the three phases of investigation -conceptual, empirical and technical- mutually inform each other.^10^

In the conceptual investigation, CSD practitioners explore the central issues as well as big questions related to the design context at hand. In the case of designing a chatbot for mental health, one could think of big challenges such as whether chatbots might degrade human friendships since people might confide in technology rather than in the people around them once the chatbots are widely available, or whether the chatbots might facilitate a future situation in which only the rich will still have access to human health care professionals. With tools such as ‘Envisioning Cards’^11^ CSD practitioners together with stakeholders can reflect on the challenges attached to the design context and subsequently select the relevant capabilities to guide them in the design process. For example, the capability of having control over one’s material and political environment together with the capability of having good health could guide CSD practitioners in designing a chatbot that presents us an alternative future situation than one in which only the rich have access to human health care professionals. Or the capability of having social affiliations with others could guide CSD practitioners in designing a chatbot that diminishes the risk that human friendships ultimately degrade.

Important to note here is that “design typically concerns products that do not yet exist” (Van de Poel 2012, p. 296). This causes an epistemological challenge, as Ibo van de Poel has pointed out (2012). This entails that CSD practitioners need knowledge both of “what constitutes well-being for users and how that well-being might be affected by new technologies”, as well as awareness “that such knowledge needs to be translated into, for example, design requirements, criteria or technical parameters that can guide the design process” (Van de Poel 2012, p. 296). Knowledge of the first aspect, on what constitutes wellbeing for users, can be explored with the “philosopher-investigator” approach (Claassen 2011) as discussed in "Capability Approach and Capability Theory" section of this article, a strategy on how this knowledge can subsequently be translated into design requirements is presented in the ‘technical investigation’ when the concept of the ‘capability hierarchy’ (Van de Poel 2013) is discussed.

Another important challenge put forward by van de Poel (2012) is the problem of aggregation, which entails that if wellbeing constitutes plural and incommensurable capabilities or values then the challenge presents itself how to aggregate these capabilities into an overall measure of wellbeing. A possible solution to this challenge is recently put forward by Cenci and Cawthorne (2020), who point out that what is necessary to circumvent the aggregation problem is “qualitative deliberative workshops based on a focus groups research design, since incommensurable values-capabilities as well as “aggregated” social/public value *naturally* emerge from small-sized and localized procedures based on face-to-face interpersonal critical discussion and deliberation” (Cenci and Cawthorne 2020, p. 27). A study by Cenci and Hussain (2019) points out, however, that “Nussbaum’s rigid and over specified list” is less suitable to facilitate this than Sen’s version of the capability approach with its “underlying procedural ethics ideal” (Cenci and Cawthorne 2020, p. 28). However, with the “philosopher-investigator” approach (Claassen 2011) that CSD practitioners are recommended to follow, as discussed earlier in "Capability Approach and Capability Theory" section of this article, there is sufficient room for the necessary critical discussion and deliberation among practitioners and stakeholders.

Now, in the specific context of the design of an AI-based therapy chatbot to help improve people's mental health, the following capabilities from Nussbaum’s list could be identified as relevant:

1. The capability of being able to live in health, both physically as well as mentally. Health, as argued for in "Capability Sensitive Design" section, can best be understood in this context as a person's ability to realize one's vital goals and the ability to exercise a cluster of basic human activities.
2. The capability of being able to experience human emotions. Being able to experience love, grieve, longing, gratitude and justified anger, and not have one's emotional development blighted by overwhelming fear and anxiety, or by traumatic events of abuse or neglect.
3. The capability of being able to have social affiliations with others. To have the social bases of self-respect and non-humiliation, and to engage in various forms of meaningful social interactions with other human beings.
4. The capability of being able to enjoy life, to play, laugh and have fun.
5. The capability of practical reason, to plan and give direction to one’s life.
6. The capability of having control over one’s material and political environment.

### Functionings

The functionings that correspond with the selected capabilities include the following: the functioning of actually being in good health, both physically as well as mentally, the functioning of actually having emotional attachments to others and not be overwhelmed by experiences of fear or anxiety that limit ones emotional development and capacities, the functioning of actually having meaningful and respectful social affiliations and engage with others in social interactions, the functioning of actually enjoying oneself and laugh and play, and the functioning of making plans and give actual direction to one’s life.

Important to note is that a technology such as the therapy chatbot should foremost be *capability* enhancing and it should ultimately be up to the user to choose whether or not to actually turn a capability into a functioning. However, a technology can steer someone in a certain direction; persuading the user to engage with the technology in a certain way and perform certain target behavior. For persuasion by technology to be ethically permissible, as I have argued elsewhere (Jacobs 2020), it is important that persuasion never (1) significantly blocks or burdens options, that (2) a person is aware of the fact that one is being intentionally influenced, and aware of the mechanisms of that influence, and (3) that the influence is in the best interests and in alignment with the personal goals of the person being influenced. If these conditions are met, then it is ethically permissible that a technology steers users in a certain direction, aiming that they actually turn certain capabilities into functionings.

### Direct and Indirect Stakeholders

In the conceptual investigation, CSD practitioners as well identify the relevant direct and indirect stakeholders involved. The direct stakeholders are those people who will interact directly with the technology or the technology's output. The indirect stakeholders are those people who won't interact with the technology directly but who might be impacted by it (Friedman and Hendry 2019). There are various methods developed in the empirical social sciences to identify direct and indirect stakeholders, it exceeds the scope of this article to discuss these methods in detail but I like to refer here to the work of Sarah Spiekermann (2015, pp. 174–175) on identifying stakeholders. Furthermore, the inputs and values of stakeholders can be gathered with the help of multiple empirical methods such as interviews, surveys, workshops or focus groups (Friedman and Hendry 2019; Cenci and Cawthorne 2020).

Direct stakeholders involved with the design of an AI-based therapy bot for mental health improvement are people dealing with mental health difficulties and are willing to make use of an AI-based therapy bot; these direct stakeholders are the end-users of the technology. Indirect stakeholders involved are e.g. health care professionals such as psychologists and psychiatrists who might have to deal with patients that are also making use of an AI-therapy bot besides their services, or with patients that initially replace their services because they turn to the therapy chatbot first. Other indirect stakeholders are: family and friends of people suffering from mental health issues that will make use of the therapy bot; health insurance companies that want to reduce the costs of regular mental health care; employers that want to prevent (long-term) sick leave or burn outs amongst employees and are therefore interested in the services of a AI-therapy bot for their employees; and governmental policy makers that are working on policies to improve public mental health and wellbeing.

Once the stakeholders are identified, they are engaged in the process of discussing, selecting and specifying the relevant capabilities and functionings for the design context at hand.

### Conversion Factors & Human Diversity

After identifying the direct and indirect stakeholders, CSD practitioners need to identify the conversion factors that are at play for these stakeholders. That is: practitioners need to get clear what the abilities of these various stakeholders are to convert *the means of the therapy chatbot* into *functionings*, since people have various abilities to transform a resource into a functioning (Robeyns 2017).

Due to limitations of space, the focus here will be solely on conversion factors of the direct stakeholders: the people who are dealing with mental health difficulties and are willing to make use of an AI-based therapy chatbot. The following conversion factors can be identified upfront as important to take into consideration^12^:

Personal conversion factors that are relevant include (1) the nature and severity of the mental illness that a person is suffering from, which might have influence on the person's receptiveness to (online) therapy; (2) whether or not, or to what degree, people are capable of expressing their (troubling) thoughts and feelings into words and write them down in conversation with a chatbot; (3) whether or not people are digitally literate enough to use a smartphone or computer and interact with an online chatbot.

Social conversion factors that are relevant include (1) whether or not there are societal norms such that people are comfortable enough to honestly speak about their (troubling) thoughts and emotions with someone, or some*thing* in the case of the therapy bot; (2) whether or not there exist social stigmas associated with certain mental illnesses that make it hard for people suffering from these conditions to speak about their experiences; (3) the size and quality of the social network that is available to a person, does the person has (many) relatives, friends, or caregivers to rely on? (4) Are certain people's voices not 'heard' or undermined on account of their illness, gender, sexuality, race, class or age, or because society lacks the concepts to name and acknowledge the (mental) symptoms someone is suffering from? In order words, are there epistemic injustices (Fricker 2007) at play towards certain (groups of) people in society?

An environmental conversion factor that is relevant includes whether there is a digital infrastructure such that people can easily make use of an online application such as an AI-therapy bot at places that they deem comfortable and private enough to use the chatbot.

By explicitly focusing on the varieties of stakeholders' conversion factors (i.e. their personal characteristics, social settings and environmental factors), CSD practitioners account for the diversity of stakeholders involved. And as already indicated previously, accounting for diversity is of crucial importance because when certain stakeholders are not brought up to a certain threshold level of a capability that has been identified as morally relevant, due to the fact that the stakeholder group lacks the required conversion factor(s) needed to turn the recourse(s) of a technology into a functioning, than the technology design not only fails these stakeholders, but might also be morally unjust.

At this point, it is worthwhile to take a step back, and recall for a moment that VSD was facing various challenges when the method is *not* complemented by an ethical theory such as the capability theory by Nussbaum, as was discussed in the introduction of this article. The reason why it is worthwhile to recall this is because two out of the three challenges that VSD was facing without complementation of an ethical theory such as Nussbaum’s capability theory, are at this point addressed.

First of all, there was the challenge that practitioners of VSD fail to make explicit their own voice in the design process, including their values and normative commitments, and thereby cause the risk that unfounded and unjustified moral authority is being claimed (Borning and Muller 2012; Jacobs and Huldtgren 2018). Although in the latest literature on VSD Friedman and Hendry (2019) state that “designers are encouraged to make their own values, as well as the project vales, explicit and transparent throughout the design process” (p. 38) it remains unclear how designers should do this. However, by indicating at the start of the design process that the moral principles that CSD practitioners are making use of are derived from Nussbaum’s capability theory, this problem is addressed. From the start of the CSD process it is now explicit that practitioners are guided by the moral principle that all human beings are equal and worthy of a life worth living -which entails that people have access to ten central capabilities- and that human diversity should be respected in technology design and (structural) injustices diminished. Now, when practitioners at the start of a new design process formulate their values in direct response to the normative input of CSD, then their voice and the normative commitments that are being made in the design process are explicit and transparent.

Secondly, VSD on its own faces the challenge of conflating facts and values. That is because the values solicited from stakeholders are taken as leading values in the VSD process without questioning whether what *is* valued by stakeholders, also *ought* to be valued (Jacobs and Huldtgren 2018; Manders-Huits 2010). VSD practitioners often assume to know what to do in a *normative sense* when knowing *empirically* what values stakeholders hold. But as myself and Huldtgren have pointed out: "even if empirical data is able to show *that* people hold certain values, it is not able to say anything about whether people *should* hold certain values" (Jacobs and Huldtgren 2018). In order to avoid the conflation of facts with values -i.e. the naturalistic fallacy- the normative input for the VSD analysis should be derived from ethical theory, as argued for by Manders-Huits (2010) and Jacobs and Huldtgren (2018). CSD addresses this problem by deriving its normative input from Nussbaum’s capability theory and her list of ten capabilities, providing CSD practitioners with normative input to argue what *ought* to be valued and thereby addressing the naturalistic fallacy. Important to note is, however, that CSD advocates the “philosopher-investigator” strategy as put forward by Claassen (2011) and discussed earlier in "Capability Approach and Capability Theory" section. To summarize, this entails that CSD practitioners will let the normative input of Nussbaum’s capability theory be influenced by the results of empirical stakeholder analyses. For example, by using the tool of “capability cards” (Steen 2016) practitioners discuss with stakeholders which capabilities from Nussbaum’s list to focus on in the technology design project and how to specify these capabilities in the design context. How the role of stakeholders exactly takes shape is discussed in more detail in the following paragraph.

Finally, a challenge that to this point remains for VSD as well as for CSD is the challenge how to make trade-offs in the case of value- or capability conflicts. This challenge will be discussed in detail in "CSD for Health & Wellbeing Technology" section.

### Empirical Investigation

The empirical investigation explores if the findings of the conceptual phase correspond with the experiences and values of the direct and indirect stakeholders. With the help of various empirical methods such as interviews, surveys or focus groups (Friedman and Hendry 2019) CSD practitioners develop an understanding of how stakeholders are experiencing current provisions and services of mental health care, what stakeholders currently value and what they are missing, what their initial impressions are of a therapy bot, and what conversion factors play a role for them. Furthermore, in conversation with the stakeholders, CSD practitioners further specify the selected capabilities, adding context-specific content to the abstract capabilities. Subsequently, based on these findings CSD practitioners can make prototypes of the envisioned technology that incorporates the results of the conceptual and empirical investigation (how this works is explained in the next paragraph). Then, after the making of the first prototype(s) there is, ideally, a second empirical investigation conducted in which the prototype is presented to the stakeholders and their assessment of the prototype is explored, i.e.: whether the stakeholders experience if the technology design sufficiently accounts for the selected capabilities, whether all relevant conversion factors are sufficiently accounted for enabling them to actually turn the technology into a functioning, and whether they experience any conflicts between valuable capabilities in the technology design. Based on these findings, the prototype is adjusted up to the point that it finds its 'ideal' form, to the extent that is feasible.

### Technical Investigation

How are the selected capabilities providing normative guidance in the actual design process, i.e.: in the *technical* part of the design phase? In other words: how do the selected capabilities affect, or even dictate, what tangible design requirements should be met in the design of an AI-based therapy chatbot to help improve people's mental health?

Van de Poel (2013) has developed the notion of *value hierarchy* in order to facilitate the translation of values into concrete design requirements for the VSD method. Following the workings of Van de Poel's value hierarchy, we can re-conceptualize the hierarchy as *capability hierarchy*, as proposed by Oosterlaken (2015a).

**Fig. 1:**
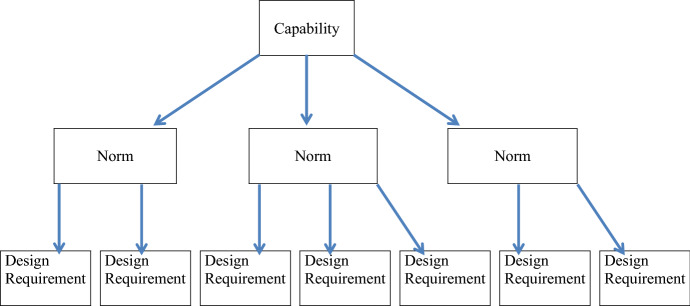
Basic outline of a capability hierarchy following the example of Van de Poel's (2013) value hierarchy

According to Van de Poel "a values hierarchy is a coherence structure that is held together by two relations" (2013, p. 254). First there is the relation of 'specification' by which values are translated into norms, and norms into design requirements. Secondly, there is the relation of 'for the sake of' by which design requirements are connected to the higher-level elements of norms and values. That is: a design requirement is fulfilled 'for the sake' of the norm, which is the higher-level element that dictates the design requirement, and the norm is fulfilled 'for the sake of' the value or capability which forms the highest-level element in the hierarchy. A basic outline of a capability hierarchy looks like this: (Fig. 1).

**Fig. 2:**
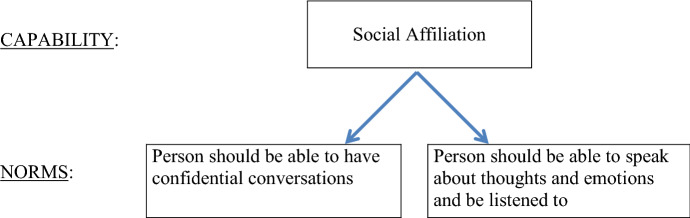
Detail of capability hierarchy

Values, or in the case of CSD: capabilities, are relevant for evaluating the worth or goodness of certain things. However, values or capabilities do not directly imply certain prescriptions or restrictions for action, Van de Poel rightly points out (2013). Norms, contrary to abstract values or capabilities, do have a prescriptive character. Thus, the aim is to get from an abstract value or capability, to a more specific norm that articulates certain prescriptions for or restrictions on action. This translation is done with the methodological tool of specification, which is often used in mid-level theories. Specification adds context- or domain-specific content to abstract values or capabilities. There are two criteria for the adequacy of translating a value or capability into norms: the norms should be an appropriate response to the value or capability, and the norms should be sufficient to properly respond to or engage with the value or capability (Van de Poel 2013). For example: the capability of 'having social affiliations that are meaningful and respectful', which is identified as a relevant capability for the design of a therapy chatbot to help improve people's mental health, can be specified by adding context-specific content such as that people suffering from mental health problems often experience an absence of people they can talk to about their thoughts and emotions and who can provide them with emotional support. The addition of this context-specific information to the abstract capability of 'having social affiliations that are meaningful and respectful' helps us to formulate more specific norms that are appropriate and sufficient responses to the capability. Such norms could be: ‘A person should be able to have confidential conversations’ and ‘A person should be able to speak about one’s thoughts and emotions and be listened to’ (Fig. 2).

The next step is to translate these norms into concrete design requirements. The norm 'A person should be able to have confidential conversations' can e.g. be translated into the design requirement that the conversations between the user and the chatbot are end-to-end encrypted so that no one other than the user has excess to the data that is shared in the conversations, thereby assuring that the conversations are confidential and private.

A design requirement derived from the second norm 'A person should be able to speak about one’s thoughts and emotions and be listened to' could be to program the chatbot in such a way that it encourages users with brief and daily 'check-ins' to ask how the user is doing and encourage them to take the time to tell their stories. For example, the chatbot Woebot sends out short daily messages asking users questions such as: 'tell me, what are you doing at the moment?' followed by an emoticon of a pencil. Or: 'got a second to reflect on what you're grateful for today?' These brief daily check-ins function as conversation starters and encourage users to take a moment to write down their thoughts and feelings.

**Fig. 3:**
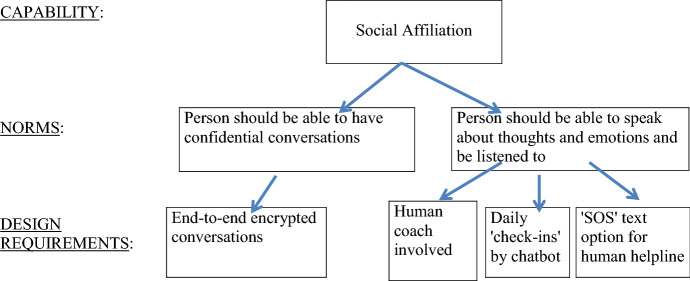
Detail of capability hierarchy II

Another design requirement derived from this norm could be that users can text 'SOS' when they want to talk to a human health care professional instead of talking to the AI-based chatbot in case of an alarming situation. Chatbots Woebot and Wysa direct users to international helplines when they type in 'SOS'. In addition, chatbot Wysa also offers users the option to subscribe for an additional fee to a personal coaching plan with a human coach (Fig. 3).

With the help of the capability hierarchy, CSD practitioners can translate the abstract capabilities into concrete design requirements. Subsequently, based on the identified design requirements, CSD practitioners then build (a) prototype(s) of the envisioned technology and empirically test with the stakeholders whether the prototype sufficiently facilitates the capabilities that are identified as important and whether the prototype accounts for the relevant conversion factors at stake for the stakeholders involved. Based on these empirical findings technical adjustments can be made.

## Challenges for CSD

The CSD framework, however, faces some challenges, namely the challenge of (1) sufficiency, (2) dealing with capability conflicts, and (3) dealing with ‘multistability’. I will briefly discuss these challenges and explain how they can best be dealt with.

A first challenge is how to determine whether a design ultimately meets the criteria of the capability hierarchy in a satisfactory way. That is: how can CSD practitioners determine whether they have accounted for all the norms derived from the selected capabilities, and subsequently whether they *sufficiently* accounted for all the design requirements that can be derived from these norms? This is a persistent issue that resurfaces in design approaches in general and there is no satisfying response to the issue of sufficiency. The most viable option for CSD is to retrace the capability hierarchy step by step and to move back and forth between the conceptual, empirical and technical investigations, making various iterations in consultation with the relevant stakeholders to see whether the conditions of the capability hierarchy are sufficiently met.

A second challenge for CSD practitioners is how to deal with conflicts between capabilities in design contexts. That is: when two or more capabilities cannot be realized at the same time in a technology design, how should CSD practitioners prioritize one capability over the other?

For example, in the design case of the AI-driven therapy chatbot for mental health the following conflict between capabilities could occur: as discussed above, the capability of social affiliation is important in this design context. The capability of social affiliation can be translated in the norm 'a person should be able to have confidential conversations', which in turn could be translated into the design requirement that the conversations between the user and the chatbot are end-to-end encrypted so that no one other than the user has excess to the data that is shared in the conversations. However, the capability of health is also selected as an important capability in this design case and the capability of health can be translated into the norm that 'users suffering from mental health issues should be guided by health care professionals that have the expertise and skills to assist them'. This norm, subsequently, can be translated into the design requirement that the data shared by the user with the chatbot should also be monitored by qualified health care professionals in order for them to provide users with adequate help in cases of crisis.

Now, a conflict arises on the level of design options: one design option favors encryption of user data, while the other design option favors monitoring user data by health care professionals. Both design options refer to different capabilities: the design option of encryption refers to the capability of affiliation, while the design option of monitoring refers back to the capability of health. The question for CSD practitioners now is: what design option to favor and thus what capability to choose above the other?

Nussbaum has been very clear on the question, stating that each of the ten capabilities on her list is important and incommensurable, and thus cannot be traded off against another. In the case a capability conflict occurs and not all capabilities can be secured, “it becomes a purely practical question what to do next” Nussbaum has stated (2006, p. 175).^13^ In line with Nussbaum’s position, Van de Poel has proposed “a solution to set thresholds for all relevant capabilities and to look for a design that reaches all these solutions” (Van de Poel in Oosterlaken 2015a, b, p. 236). Let me elaborate on this solution. As the example above shows, a conflict occurs on the level of design options that in turn refer to norms, which refer to capabilities. Important to note is that the trade-off here should be made on the level of design options and this is thus the level to look for a solution. This is important to keep in mind since one can be convinced that a normative decision needs to be made on the level of capabilities, namely what capability needs to be traded off for the other, while a capability conflict can often "be tackled by means of technical innovations", van den Hoven et al. (2012, p. 144) point out. Capability conflicts may very well have creative design solutions that enable us to expand *all* capabilities concerned rather than to make a trade-off between capabilities (Oosterlaken 2015a).

The conflict could be dealt with by various design solutions: think for instance of a design solution that ensures encrypted data-sharing while also offering users a 'red panic button' that users can press when they are in need of a human health professional to assist them in case of crisis. Another design solution could be to offer users encrypted data-sharing while also ask them at the start of using the chatbot to fill in an emergency contact that can be contacted in the case there is good reason to think the user poses a danger for themself or others. Yet another design solution could be to have users take a self-assessment test at the start of their use of the chatbot to determine the severity of their mental health issues and to determine whether or not the user is at an acceptable level to use the chatbot or whether a user’s issues are so severe that one needs to be directed to a human health care professional. Thus, what CSD practitioners should do is come up with various design solutions to a capability conflict, which they subsequently pilot with the help of design prototypes among stakeholders in a second or third empirical iteration to find out what design solutions works best.

However, capability conflicts can present themselves also in different ways: not only can a capability conflict occur between two capabilities, there is also the possibility that a conflict occurs due to interpreting the same capability in two different ways. That is: various stakeholders could specify the same capability in different ways, seemingly leading to incompatible understandings of that capability and subsequently leading to different norms and design requirements that are derived from that understanding of the capability.

For example, as discussed earlier in this article, the capability of health can be specified in various ways. That is: the context and domain-specific content that can be added to the capability of health can differ greatly based upon what conception of health stakeholders adhere to. Thus, one stakeholder group, e.g. the designers of the therapy chatbot, could specify the capability of health using Nordenfelt's conception (1986) as people's ability to realize their vital goals. While the stakeholder group of health care professionals could specify the capability of health by Christopher Boorse's conception (1975) as statistical normality of functioning and the absence of disease.

The second stakeholder group, that adheres to the conception of the capability of health as the absence of disease, could derive from that conceptualization the norm that people should be able to live in absence of mental illnesses such as depression. While the first stakeholder group derives the norm that people should be able to undertake activities that are valuable to them, with or without the presence of a mental illness such as depression. Subsequently, the design requirements that follow from these different norms could point into conflicting directions, i.e.: either into the direction of curing disease, or into the direction of enabling people with (or without) disease to realize their abilities.

In such cases, it is important that CSD practitioners return to the process of specification, which forms the heart of the capability hierarchy. As already indicated, the process of specification consists of reducing the indeterminacy of an abstract capability into less abstract norms and subsequently into concrete design requirements. Reducing indeterminacy and narrowing the scope of capabilities and norms comes down to "spelling out where, when, why, how, by what means, to whom, or by whom" (Richardson 1990, p. 289) an action is to be done or avoided. In jointly spelling out the where, when, why, how, by what means, to whom, or by whom something needs to be done or avoided, stakeholders that (initially) have different conceptions of a capability can come to an agreement via a process of deliberation on how to specify the abstract capability at hand, and subsequently into what norm(s) the capability can best be translated. In the case that differences remain on how to specify a capability, agreement can be sought on the level of norms and ultimately on the level of design requirements by looking for technical solutions that expand all conceptualizations of the capability and facilitate the various normative prescriptions that are identified.

Thus, by going through the process of specification that forms the core of the capability hierarchy, stakeholders are jointly deliberating on the relevant context- and domain-specific content that should be added to the abstract capabilities, with the aim to ultimately come to a joint solution on the design level. And thus again; capability conflicts (arising either between two capabilities or within the specification of one and the same capability by different stakeholders) may very well have creative design solutions that enable us to expand *all* (conceptions of) capabilities concerned, rather than to make a trade-off.

A last perpetual challenge for technology assessment in general is how to deal with unforeseen consequences of technology design. Anders Albrechtslund (2007) calls this the 'positivist problem', pointing out that in many design approaches the default position is "that the design of a technology will -more or less- correspond with the use of technology and that this relation does not pose a problem" (2007, p. 68). However, the relation between design contexts and user's practice is often complex and unpredictable: technologies are frequently used in ways different than initially intended and technologies "can be conceived of differently according to cultural, historical and social contexts" (2007, p. 68). Recent research, for example, has shown that people are "more compliant when a robot asks them to do something as compared with a person" (Fiske and colleagues 2019, p. 6). This could lead to the unforeseen and unwanted consequence that people share or do things that they otherwise would (and possibly should) not do. In the case of therapy chatbots, this could lead to the situation of people (over-)sharing personal and sensitive information without them properly realizing that the information is stored and used by the company behind the chatbot, and possibly even shared with third parties.

Ihde (1993) has pointed out that a defining characteristic of technology is its ‘multistability’. This means that technology does not have a pre-existing essence or basic meaning apart from the use contexts it enters into. This defining characteristic of technology is what Ihde calls 'multistability'. The challenge design approaches in general face, and thus also CSD, is how to acknowledge and account for the multistability of technology. There is a fundamental openness implied in the concept of multistability, Albrechtslund (2007) points out, and this creates a difficulty for technology designers and developers to come up with a comprehensive list of potential human-technology relations and use contexts. The challenge is to "imagine potential use contexts and the ethical scenarios they create […] and to envision as many multistabilities as possible while designing technology in order to anticipate future ethical problems and dilemmas" (Albrechtslund 2007, p. 70). What CSD practitioners should do in order to meet the challenge of the positivist problem is to acknowledge technology's multistability and the unpredictability of contexts of use, while at the same time account for active stimulation of "creative thinking to imagine the near-unimaginable" (Albrechtslund 2007, p. 70) in all three investigations of the CSD process.

## Concluding Remarks

This article presented the framework of Capability Sensitive Design, which consists of a merging of VSD with Nussbaum's capability theory. Various reasons have been given for why CSD can contribute to the normative assessment of technology design in general, and to technology design particularly for health and wellbeing. Subsequently, the general workings of CSD have been demonstrated by applying it to a hypothetical design case of a therapy chatbot. The article has shown how CSD addresses VSD’s challenge that the voice of practitioners is obscured and thereby causing the risk that unfounded moral authority is being claimed, since the normative input in CSD is explicitly derived from the core claims of Nussbaum's capability theory and her list of ten capabilities. Furthermore, CSD has shown to be able to address VSD's challenge that stakeholder values are taken as leading values in the design process without questioning whether what *is* valued by stakeholders also *ought* to be valued, since within CSD it is normatively argued for that what *ought* to be valued are the selected capabilities based upon the normative core claims of Nussbaum's capability theory. Although the challenge of conflicting capabilities or values remains for both VSD and CSD, a strategy has been presented for how CSD could deal with occurring capability conflicts. To conclude, this article has presented the framework of CSD and illustrated its general workings, its merits over the standard VSD approach, and most importantly: what a new technology design for health and wellbeing such as a therapy chatbot would look like when attention is paid to capabilities right from the start of the design process. Now it is time for future empirical work on the application of CSD to determine its merits and limits in practice.

## Footnotes

1 Present-day examples of technology design in which values such as fairness or equality were undermined include racial discrimination in facial recognition software and algorithmic bias in recruiting software tools, while the app I Reveal My Attributes (IRMA) is an example of a software design that explicitly supports the value of privacy.

2 Batya Friedman and David Hendry indicate in their book *Value Sensitive Design, Shaping Technology With Moral Imagination* (2019) that “value sensitive design resists overarching normative directives, for example, about which ethical theory to adhere to” (p. 8). The strength of VSD without adherence to an ethical theory, as an anonymous reviewer pointed out, partly lies in its simplicity, versatility and adaptability. However, as this article will hopefully convincingly show, there are various crucial advantages for VSD when complemented by an ethical theory. Friedman and Hendry seem to recognize this when they write that: “mechanisms exist through which normative elements can be embedded in VSD. For example, a particular ethical theory could be employed in a given project, as Aimee van Wynsberghe (2013) did in her application of care ethics to robotic assistance in health care” (2019, p. 8).

3 The distinction between the capability *approach* and capability *theories* is elaborated on in "Introduction" section.

4 How exactly this can take shape is discussed at the end of this section.

5 Harm is understood here as experiencing racial discrimination, and of course the harm of not being able to properly wash your hands.

6 Important to keep in mind, as rightfully pointed out by an anonymous reviewer, is that values may change; new values may emerge, the priority or relative importance of a value may change, the way in which a value is conceptualized, as well as how a value is specified may change over time and depending on context. In certain times e.g. homosexuality was (and in certain contexts still is) condemned. The value of sexual expression in those times and contexts has a different meaning, and the corresponding capability and functionings (to sexual expression) may not have not have been considered acceptable.

7 By no means is this a fully comprehensive CSD analysis of such a therapy chatbot, instead it is a basic outline of a CSD analysis in order to show the general workings of CSD and to illustrate the merits that CSD can have over VSD as a design approach, especially in the case of design for health and well-being technology.

8 One could point out -as an anonymous reviewer rightfully did- that this is essentially a policy problem that comes down to the question whether designers should be tasked to make cheaper mental health tools or whether policy makers should increase funding for mental health? The value sensitive-alternative approach developed by Sarah Spiekermann (2015) invites us to think up valuable alternatives to standard technology and calls us to forgo the use of ethically problematic systems and to actively seek ethical alternatives to create value. Although this is indeed a crucial issue to consider at the start of each design process, in the specific case of the design of a mental health chatbot there are significant benefits for users of the technology, such as its 24/7 availability at any place with an internet connection and its ability to avoid stigmatization attached to frequenting a human mental health care professional. Therefore, it can be deemed worthwhile to explore a CSD approach to the design of such a mental health chatbot.

9 Please recall that Nussbaum’s list is open-ended; it is not definite but open for discussion and revision (Nussbaum 2000, p. 77) thus it is possible that CSD practitioners and stakeholders agree to design for a capability that is not on the list.

10 A risk rightfully pointed out by an anonymous reviewer is that once a selection of capabilities is made, CSD practitioners and stakeholders might overlook the importance of one or more capabilities that later on in the design process turn out to be of importance as well. This risk can be minimized, however, by following the iterative and integrative methodology of CSD that emphasizes the importance of moving back and forth between the conceptual, empirical and technical investigations and have findings from a later phase in the design process influence earlier findings and vice versa.

11 The Envisioning Cards are a toolkit developed by (Friedman et al. 2011) that can be used for ideation, co-design, heuristic critique, evaluation, and other purposes (Friedman and Hendry 2019, p. 85).

12 By no means is this an exhaustive list of all conversion factors that should be taken into consideration since conversion factors depend greatly on context. Thus, more relevant conversion factors will be discovered in the empirical investigation when empirical research is being conducted among stakeholders and prototype testing takes place.

13 As Robeyns (2016) has pointed out, “it is striking that very few proposals on selecting and weighing or aggregating have been worked out by philosophers based on foundational work on ethical theory.” However, it exceeds the scope of this article to provide theoretical grounding that resolves the issue of conflicting capabilities.
